# The *Staphylococcus aureus* extracellular matrix protein (Emp) has a fibrous structure and binds to different extracellular matrices

**DOI:** 10.1038/s41598-017-14168-4

**Published:** 2017-10-20

**Authors:** Jennifer Geraci, Svetlana Neubauer, Christine Pöllath, Uwe Hansen, Fabio Rizzo, Christoph Krafft, Martin Westermann, Muzaffar Hussain, Georg Peters, Mathias W. Pletz, Bettina Löffler, Oliwia Makarewicz, Lorena Tuchscherr

**Affiliations:** 1 Institute of Medical Microbiology, Jena University Hospital, Jena, Germany; 2 Center for Infectious Diseases and Infection Control, Jena University Hospital, Jena, Germany; 3 Center for Sepsis Control and Care (CSCC), Jena University Hospital, Jena, Germany; 4 InfectoGnostics Research Campus, Jena, Germany; 5 Institute of Medical Microbiology, Münster University Hospital, Münster, Germany; 6 Institute of Experimental Musculoskeletal Medicine, Münster University Hospital, Münster, Germany; 7 Institute of Molecular Science and Technologies (ISTM-CNR), Milano, Italy; 8 Organic Chemistry Institute and CeNTech, Westfälische Wilhelms-Universität Münster, Münster, Germany; 9 Leibnitz Institute for photon technologies, Jena, Germany; 10 Center for electron microscopy Jena University Hospital, Jena, Germany

## Abstract

The extracellular matrix protein Emp of *Staphylococcus aureus* is a secreted adhesin that mediates interactions between the bacterial surface and extracellular host structures. However, its structure and role in staphylococcal pathogenesis remain unknown. Using multidisciplinary approaches, including circular dichroism (CD) and Fourier transform infrared (FTIR) spectroscopy, transmission electron (TEM) and immunogold transmission electron microscopy, functional ELISA assays and *in silico* techniques, we characterized the Emp protein. We demonstrated that Emp and its truncated forms bind to suprastructures in human skin, cartilage or bone, among which binding activity seems to be higher for skin compounds. The binding domain is located in the C-terminal part of the protein. CD spectroscopy revealed high contents of β-sheets (39.58%) and natively disordered structures (41.2%), and TEM suggested a fibrous structure consisting of Emp polymers. The N-terminus seems to be essential for polymerization. Due to the uncommonly high histidine content, we suggest that Emp represents a novel type of histidine-rich protein sharing structural similarities to leucine-rich repeats proteins as predicted by the I-TASSER algorithm. These new findings suggest a role of Emp in infections of deeper tissue and open new possibilities for the development of novel therapeutic strategies.

## Introduction

*Staphylococcus aureus* is an important human pathogen that can cause serious invasive and persistent diseases, such as osteomyelitis or deep soft tissue infections^1^. Adhesion to the extracellular matrix of the host tissue is a critical step in *S*. *aureus* pathogenesis. This process is essentially mediated by adhesins, which are staphylococcal proteins that undergo strong interactions with the extracellular matrix. Adhesins can be divided into proteins that are covalently bound to bacterial cell wall peptidoglycans (microbial surface components recognizing adhesive matrix molecules, MSCRAMMs)^2–5^ and proteins that are only secreted but that re-bind to the bacterial cell surface (secretable expanded repertoire adhesive molecules, SERAMs)^6^. Fibronectin binding proteins (FnBPs) and collagen binding protein (Cna) belong to the group of MSCRAMMs, and their roles in bacterial adhesion to host structures and host cell invasion are well established^3,7,8^. The extracellular adhesion protein (Eap) and the extracellular matrix binding protein (Emp) are anchorless proteins and belong to the group of SERAMs^9,10^. Both proteins are essentially regulated by Sae, but their functions in bacterial adhesion remain unclear^11^. Whereas MSCRAMMs are mainly expressed in the exponential bacterial growth phase, Eap and Emp are expressed in the stationary growth phase and could therefore play roles in later infection processes^10^. Eap and Emp are not only found in bacterial supernatants but also efficiently bind to the bacterial wall via a neutral phosphatase or other unknown bacterial surface molecules^12^. PCR analysis revealed that the *eap* and *emp* genes are present in almost all *S*. *aureus* isolates, whereas they have not been found in any *S*. *epidermidis* isolates^10,13^.

The extracellular matrix (ECM) is composed of a variety of proteins and proteoglycans and glycoproteins that are secreted locally and assembled into an organized meshwork in close association with the surface of the cell that produced them^14^. Emp and Eap can interact with various ECM proteins, such as fibronectin, fibrinogen and some collagens^9,10,15^. However, Hansen *et al*. have demonstrated that the binding of Eap and Emp to extracellular proteins is strongly dependent on their incorporation into suprastructures^16^. For example, there is a striking binding specificity of Eap to different collagen types and structures. Collagen I, a major component of banded fibrils in skin, is a binding substrate in monomolecular form, but it is almost unrecognized by Eap when incorporated into banded fibrils^16^. This phenomenon can be explained by the aggregation of matrix macromolecules *in situ* into suprastructures such as fibrils, microfibrils, filaments, and networks. Furthermore, matrix suprastructures are usually composed of several molecular species, and their composition can be complexed with non-collagenous macromolecules attached to some fibril surfaces, forming a defined three-dimensional structure^17^. Conversely, the structure and function of Emp remain unknown. Surface plasmon resonance (SPR) spectroscopy has shown that Emp binds to different ECM components^10^. However, the structural adaptations and biochemical features of Emp that allow specific interactions with so many different ligands remain largely unexplored^10^. Kalinka *et al*. observed an increase in Emp expression in chronic osteomyelitis staphylococcal isolates^18^. Moreover, Emp is required for abscess formation and persistence^19^. Additionally, Emp is associated with endovascular disease^6^ and plays an important role in low-iron-induced biofilm formation^20^.

**Figure 1:**
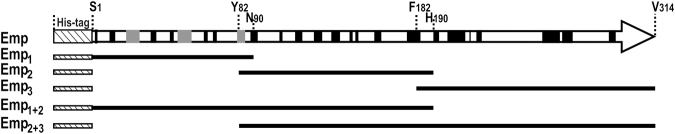
Schematic illustration of the cloning strategy used for the Emp forms of *S*. *aureus* and the predicted secondary structure. The arrow indicates the primary structure of the His-tagged fusion products of the full-length Emp protein of 314 amino acids. The His-tag (shaded box) was excluded from continuous numbering of the protein sequence. The predicted secondary structure elements within the arrow are indicated as filled boxes: black for β-sheets; grey for α-helices. The black lines below the arrow indicate the different truncated Emp fragments, which all were fused to the His-tag (grey lines).

The conferring gene *emp* (1,023 nucleotides) encodes an extracellular mature protein with a calculated molecular mass of 35.5 kDa^10^. We blasted the Emp amino acid sequence against the database to identify relatives and investigated its structural properties by circular dichroism (CD), Fourier transform infrared (FTIR) spectroscopy, and transmission electron microscopy (TEM). The polydispersity was analysed using Nanosizer measurements. Furthermore, the role of individual Emp fragments (Fig. 1) and their interactions with suprastructures in human skin, cartilage or bone were studied in *in vitro* binding assays. Finally, a possible protein structure was predicted by I-TASSER. These results provide the first structural characterization of Emp, contributing to our understanding of the role of this protein in staphylococcal pathogenesis.

## Results and Discussion

### Emp is not related to any other protein family

**Figure 2:**
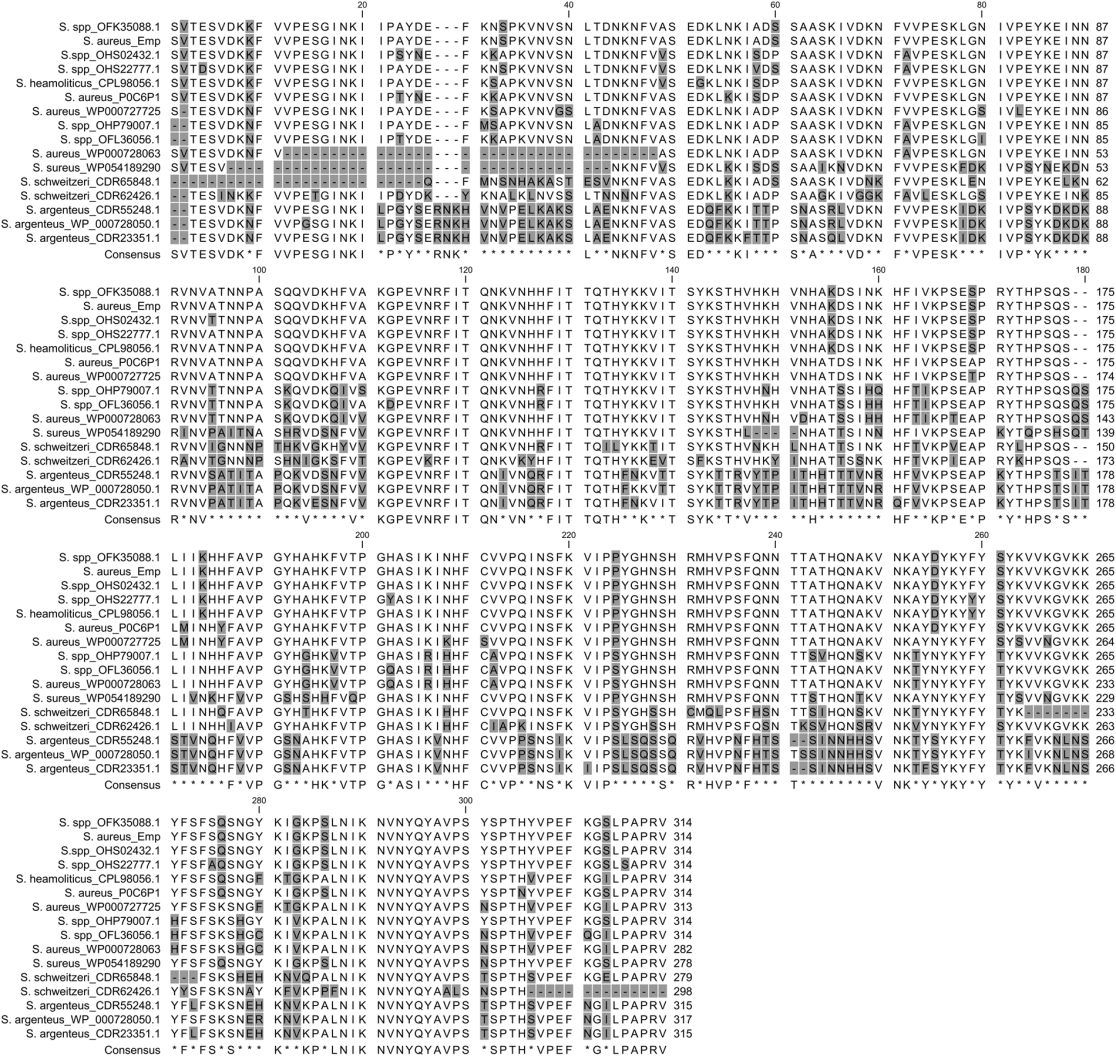
Multiple alignment of Emp proteins from different *Staphylococcus* species. Only selected *S*. *aureus* Emp proteins that showed differences in their sequences were chosen. Similarly, only those Emp protein sequences of *S*. spp, *S*. *haemolyticus*, *S*. *schweitzeri* and *S*. *argenteus* were selected that showed different identity scores in the blastp analysis.

The blastp analysis of the Emp amino acid sequence did not reveal any relationship to other known or hypothetical proteins in the database, except for other Emp forms of a few *Staphylococcus* spp. (Fig. 2). Hundreds of Emp sequences were found in the protein database within various *S*. *aureus* strains, and the sequence identity to our Emp protein was almost higher than 90% (data not shown). Excluding the *S*. *aureus* from the blastp analysis, additional Emp proteins were found in other *Staphylococcus* species. High amino acid sequence similarities were also found to various *Staphylococcus* spp. strains (identity and positivity 90–100%) and *S*. *heamoliticus* (identity and positivity 92%). Whereas Emp proteins of *S*. *schweitzeri* and *S*. *argenteus* showed lower sequence similarities than *S*. *aureus* (identity 62–75% / positivity 74–84%, and identity 61–62% / positivity 73–75%, respectively), Emp of *S*. *argenteus* showed the strongest differences in sequence with the longest phylogenetic distance from the other species (Supplementary Figure S1). Our results indicate that Emp or analogous proteins are not present in poorly invasive and low-pathogenic staphylococci species like *S*. *epidermidis*^21^.

The most extraordinary characteristic of the Emp proteins is their high content of histidines. Based on multiple alignments, at least 8 strongly conserved histidines were found in all aligned species that were primarily located within the core region of the protein (approximately from residue 123 to 240) (Fig. 2). Additional histidine residues seemed to be conserved in *S*. *aureus*, *S*. *heamoliticus*, *S*. *schweitzeri* and related species (up to 7), whereas some other His residues were conserved in *S*. *argenteus* (up to 5), indicating possible differences in functionality or substrate specificity.

Histidine-rich proteins (HRPs) have been observed in many prokaryotic and eukaryotic organisms but are not phylogenetically related to each other and do not form a structural superfamily. Due to the chemical properties of histidine, which play multiple roles in inter- and intramolecular protein interactions, they also exhibit different functions^22^. The HRPs known as filaggrins in mammals interact with the keratin cytoskeleton of the epidermis to form insoluble microfibers in the stratum corneum responsible for skin barrier functions^23^. In *Plasmodium falciparum*, the HRP-2 protein is a virulence factor that binds to the cytoskeletal components actin and phosphatidylinositol 4,5-bisphosphate^24^. Other HRPs, such as the Hpn of *Helicobacter pylori*, are responsible for Ni^2+^ storage and detoxification^25^. Moreover, human histidine-rich glycoproteins (HRGs) interact with many ligands, such as Zn^2+^, heme, fibrinogen, IgG or the complement system. HRG is involved in different processes associated with tissue injury and tumor growth to regulate cell adhesion and migration, fibrinolysis and coagulation, and the immune response^26^. Similarly, Emp binds to proteins such as fibronectin, fibrinogen and some collagens^9,10,15^; therefore, we suggest that Emp is a novel bacterial HRP.

The Emp was divided in three different constructs to be analysed (Fig. 1). The Emp_1_ is localized at the N-terminal, followed by Emp_2_ and Emp_3_ at the C-terminal. Combination of Emp_1_ and Emp_2_ was named Emp_1+2_ as well as the combination between Emp_2_ and Emp_3_ was named Emp_2+3_. The unique strongly conserved methionine (M_229_) and cysteine (C_208_) residues are located at the C-terminus of Emp_3_. For all Emp fragments, high isoelectric points (IEPs) were predicted, with an IEP of 9.99 for full-length Emp_FL_, an IEP of 8.89 for Emp_1_, an IEP of 10.38 for Emp_2_ and an IEP of 10.04 for Emp_3_. The high IEPs of fragments Emp_2_ and Emp_3_ (positively charged) suggest that those fragments might be primarily involved in interactions with negatively charged compounds or polymers.

Based on these protein sequence alignments, the most conserved region of Emp seems to be located at the C-terminus (residues 284 to 314), indicating that the C-terminus might also play a crucial role in Emp function, folding or polymerization.

### Emp binds preferentially to skin and cartilage ECMs

**Figure 3:**
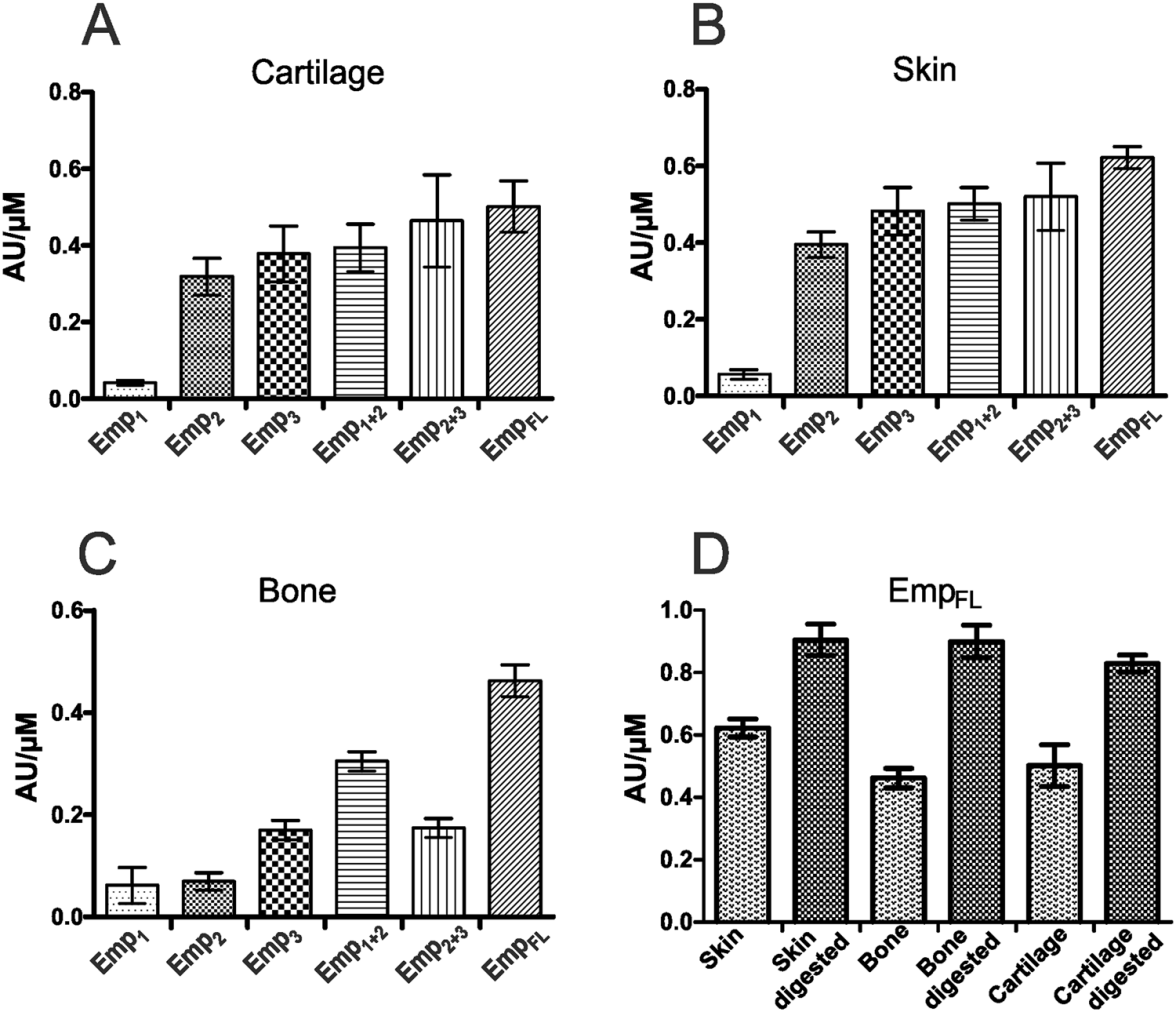
The binding specificities of Emp_FL_ and Emp fragments to different ECMs determined by ELISA assays: cartilage (**A**), skin (**B**), bone (**C**). (**D**) Binding of Emp_FL_ to enzymatically digested ECMs in comparison to non-digested ECMs. The binding specificity was expressed as absorption units (AU) per µM protein. The ECMs were immobilized on ELISA plates at saturated concentrations and were exposed to 1 µM concentrations of Emp forms.

To investigate the capacity of Emp to interact with host ECMs, the soluble recombinant protein forms Emp_FL_, Emp_1_, Emp_2_, Emp_3_, Emp_2+3_ and Emp_1+2_ were assessed for binding to human cartilage, skin and bone, which were homogenized from the target tissues and immobilized on ELISA plates as it was described in material and methods. The Emp_FL_ and all Emp forms, excluding Emp_1_, bound to skin and cartilage in a similar manner (Fig. 3A and B), whereas bone material was preferably bound by Emp_FL_, followed by Emp_1+2_ and, to similar extents, Emp_3_ and Emp_2+3_. Emp_1_ and Emp_2_ showed the lowest binding activity to the bone material (Fig. 3C). The specificity of the target might therefore depend on the C-terminal sequence because variations seemed to modulate the binding properties. Interactions between Emp and ECMs were higher with the C-terminal part of the protein (Emp_2_ and Emp_3_) but were almost absent within the N-terminus (Emp_1_), suggesting that Emp might consist of two domains: the C-terminal domain, which is responsible for selective binding to target structures; and the N-terminal domain, the function of which is not related to target interactions.

The binding activity of Emp_FL_ was compared between enzymatically digested and non-digested suprastructures (Fig. 3D). Thereby, Emp bound more efficiently to digested material, which offers more binding sites following the enzymatic release of matrix components.

**Figure 4:**
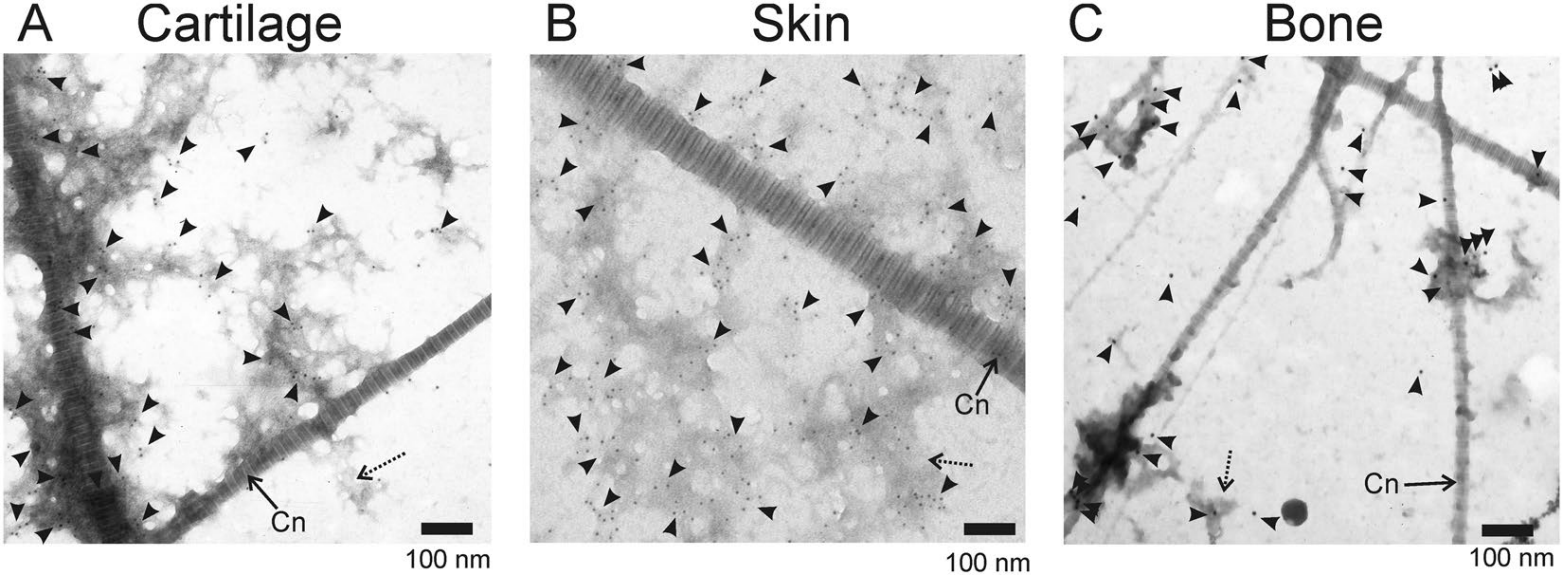
Immunogold electron microscopic images of the digested ECMs exposed to Emp_FL_. The Emp proteins are visualized by antibodies against Emp and secondary gold-labelled antibodies visible as dark spherical particles (indicated by arrowheads) located predominantly in the destroyed ECM structural formations (dashed arrows). The fibers of extracellular collagen (Cn) are indicated by arrows. The scale bars indicate 100 nm.

The specific binding of Emp to ECMs was analysed by immunogold electron microscopy (Fig. 4). The ECMs were and immobilized to the formvar/carbon coated nickel grids, enzymatically digested and exposed to Emp_FL_. As controls, ECMs were treated with PBS. Subsequently, the grids were incubated with polyclonal rabbit antibodies against Emp and anti-rabbit IgG conjugated to colloidal gold particles. Non-specific binding was not detected in negative controls without Emp or anti-Emp treatment (data not shown). Emp_FL_ adhered to all tested matrices but was primarily found in the enzymatic-treated regions because more epitopes are available (indicated by arrowheads in Fig. 4). These experiments also showed that more Emp particles were found in cartilage and skin matrix compartments than in the bone matrix.

These results suggested that Emp preferably interacts with matrix components usually buried within matrix suprastructures which becomes available after proteolytic treatment. This situation might take place during infection, when cytotoxic factors of pathogens and the host immune system damages most host matrices (i.e., inflammation) and internal matrix components are exposed. Emp might be used by *S*. *aureus* to bind to these structures for internalization into different host cells and tissues^27–29^.

### Emp binds host tissue components

**Figure 5:**
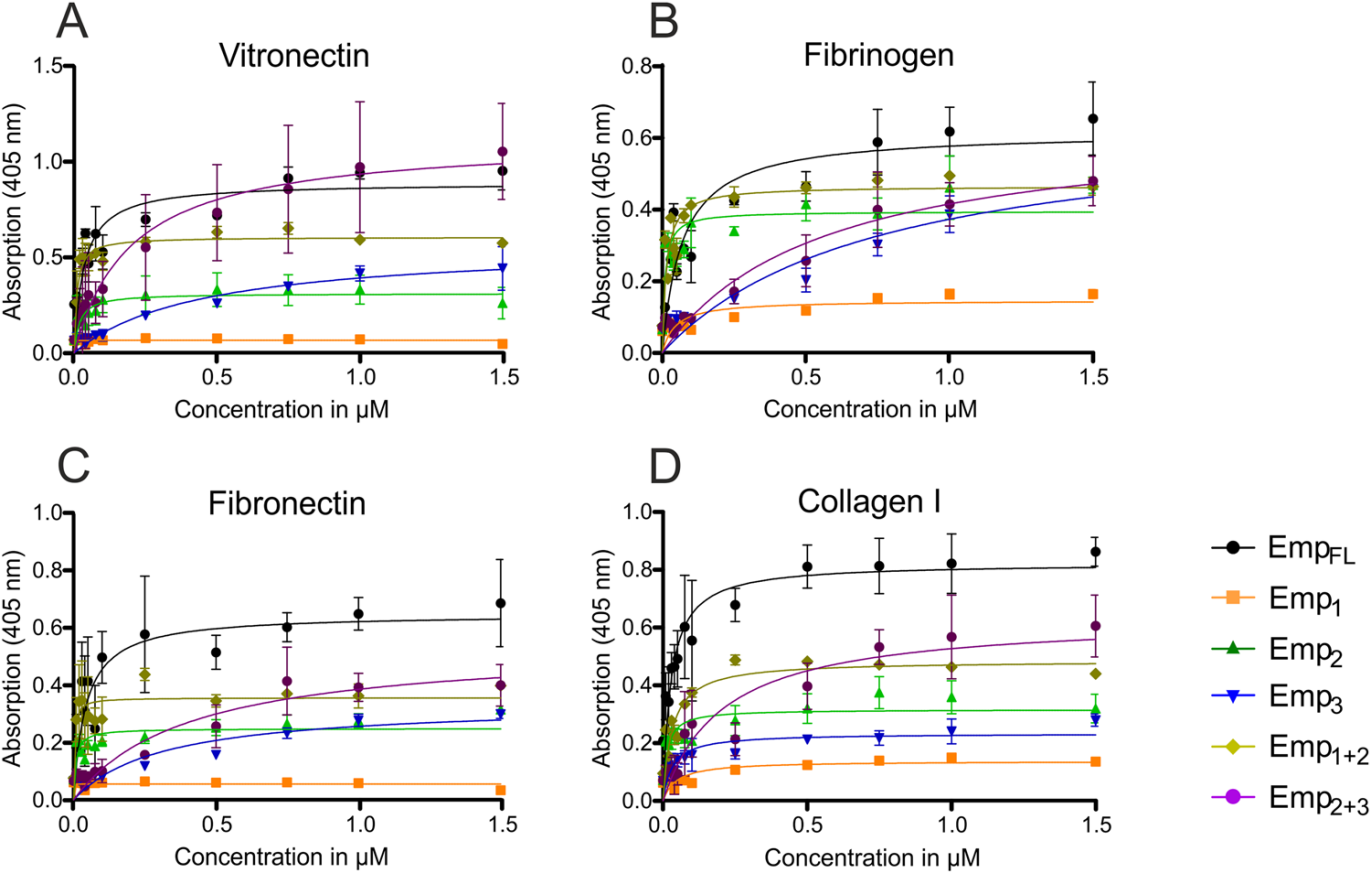
Binding kinetics of the Emp forms to different matrix components: vitronectin (**A**), fibrinogen (**B**), fibronectin (**C**) and collagen I (**D**). The legend (in **D**) corresponds to all diagrams. The curves represent the average values with the standard deviation of at least three independent measurements.

*S*. *aureus* adheres to ECM substrates and eukaryotic cells to initiate invasive infection^15,30^. Each ECM contains a special arrangement of components like fibrinogen (Fg), fibronectin (Fn), vitronectin (Vn) and collagen I (Cn I), which form a three-dimensional structural complex^30^. ELISA was performed to study the specific interactions of the Emp forms and either Fg, Fn, Vn or Cn I. All matrix components were immobilized on ELISA plates and incubated with increasing concentrations of Emp_FL_ and the different Emp forms. The mean equilibrium dissociation constant (K_D_), which represents a measure of affinity, was calculated from the steady-state binding curves of the different Emp forms at different ligand concentrations (Fig. 5). Thereby, the lower the K_D_ value indicates a higher affinity.

Emp_FL_ and all Emp forms, except for Emp_1_, bound to all components in a concentration-dependent manner (Fig. 5). Although the K_D_ values for Emp_1_ binding to Fg and Cn I could be calculated, it remained an artefact of the assay because the maximal signal was very low compared with the other Emp forms, indicating that Emp did not interact with the compounds. The affinity of the other Emp forms varied strongly depending on the Emp fragment and the substrate.

The K_D_ values of Emp_FL_ were 27 nM for Cn I, 34 nM for Vn, 43 nM for Fn, and 70 nM for Fg (Table 1). In previous surface plasmon resonance (SPR) experiments, similar results were obtained for these ligands^10^. However, the protein was immobilized on the chip surface and the ligands were flushed through the surface. The K_D_ values obtained were: 21 nM for Fn and 91 nM for Fg, but even much lower K_D_ values were observed for Vn (0.122 nM however, a molecular mass of 1200 kDa was assumed that might cause the difference)^10^. The K_D_ for Cn I has not been calculated due to unknown molecular mass of the Cn-polymer but the SPR sensograms indicated also high affinity to Emp_FL_. These results suggest stronger binding of Emp to Cn I and Vn than to Fg and Fn. According to these results, Emp has an affinity similar to Eap for host components of ECMs^16,31^. To investigate the region responsible for the binding activity of Emp, the different Emp forms were evaluated. Emp_2_ and Emp_1+2_ showed the lowest K_D_ values, indicating the highest affinity to all tested host components with a tendency towards a higher affinity to Fg and Fn. Because Emp_1_ showed nearly no activity to the matrix components, it can be assumed that most of the amino acid residues involved in the binding of Emp to host components must be located in the central part between residues 100 to 200 (within Emp_2_). The C-terminal part of Emp (Emp_3_) showed relatively high K_D_ values for all ligands but not for Cn I, suggesting that this Emp part do not participate in the ligand-binding process. Interestingly, the presence of the C-terminus reduced the affinity of Emp_2+3_ for all ligands, suggesting that the C-terminus might be even necessary for the dissociation of Emp from host components.

**Table 1:** Binding affinities of the Emp protein forms to various substrates, expressed as dissociation constants (K_D_) in nM.

Substrate	Emp_FL_	Emp_1_	Emp_2_	Emp_3_	Emp_1+2_	Emp_2+3_
Vn	34 ± 6	/	18 ± 4	449 ± 103	9 ± 3	200 ± 50
Fg	70 ± 1	44 ± 12	9 ± 2.6	756 ± 224	14 ± 3	556 ± 115
Fn	43 ± 11	/	8 ± 3	311 ± 79	3 ± 2	404 ± 95
Cn l	27 ± 5	47 ± 12	13 ± 6	32 ± 7	31 ± 5	207 ± 44

/ = no binding.

### The secondary structure of Emp is dominated by β-sheets and natively disordered structures

The predicted secondary structure of Emp revealed a high proportion of β-sheets; only 3 α-helices have been predicted in the N-terminal fragment, Emp_1_. Most of the β-sheets seem to be separated by longer sequences that can be expected to form unstructured turns. To confirm the predicted secondary Emp structure, two methods have been applied that demand different light spectra and optical properties of the protein: FTIR and CD spectroscopy.

Using FTIR spectroscopy, the amide I bands in the IR spectra of Emp_FL_ were compared to the amide I bands of concanavalin A (14 β-sheets), myoglobin (8 α-helices), lysozyme (28% α-helices, 12% β-sheets and 60% other structures) (Supplementary Figure S2). It is well accepted that the amide I band depends on the secondary structure of proteins and can be used to estimate secondary structures^32,33^. The amide I band of the Emp protein showed a higher maximum near 1655cm^−1^, a low wavenumber slope similar to lysozyme and an unusually extended high wavenumber shoulder. Thus, the spectra could not be reliably evaluated. The deconvolution routine provides reasonable results only if the reference proteins have similar structures to the unknown protein^33,34^. However, this is not the case for the Emp protein: the broad high wavenumber shoulder in its amide I band has not been observed in IR spectra of common reference proteins, indicating an unusual and unknown conformation.

Because the FTIR analysis remained unsatisfactory, CD spectroscopy was conducted. CD spectroscopy is based on the optical activity of chiral residues that differentially absorb circular polarized light depending on the circular polarization direction^35^. Because the specific peptide bonds absorb in near-ultraviolet (UV) light (approximately 180 nm to 240 nm), the experiments were performed in NaF-containing buffer instead of NaCl, which interferes with the signal.

Based on the CD spectra (Supplementary Figure S3), proportions of 39.58% of β-sheets and 41.2% of natively disordered structures, but only 4.8% of α-helices and 13.75% of α-turns, were predicted for Emp_FL_, which confirmed the *in situ* prediction. A more detailed evaluation of the CD data further indicated that most of the helical structures were rather distorted (α_D_ = 4.58%) than regular (α_R_ = 0.23%), while the β- sheets seemed to exhibit predominantly regular conformations (β_R_ = 25.95% vs β_D_ = 13.63%). As distorted helices, all helical structures of less than four residues are assigned^36^. The deconvolution offers additional information about the averaged lengths and segment numbers of the secondary structures. The estimated segment numbers were 1.25 per 100 residues for the α-helices and 6.78 per 100 residues for the β-sheets. The predicted average lengths per segment were 3.98 residues per α-helix and 5.88 residues per β-sheet. The normalized root mean square deviation (NRMSD) fit parameter, which is a measure of the difference between the experimental ellipticities and back-calculated spectra for the derived structure based on the reference databases, was only 0.49 due to the strong scattering of the signals below 200 nm. The noisy CD data below 200 nm can be a sign of protein aggregation suggesting that Emp forms a complex quaternary structure^37^.

### The tertiary structure of Emp indicates a fibrous structure

The idea of a fibrous structure of Emp was supported by the observation that the attempt to purify mono-dispersed Emp_FL_ for structural analyses by preparative size exclusion FPLC, using both Sephadex 75 and Sephadex 200 columns, failed because we could not separate single peaks (data not shown). The protein yielded a plateau that indicated a poly-dispersed solution of polymers of different lengths. It is also possible that the protein interacted with the matrix due its adhesive characteristics, hampering the separation.

**Figure 6:**
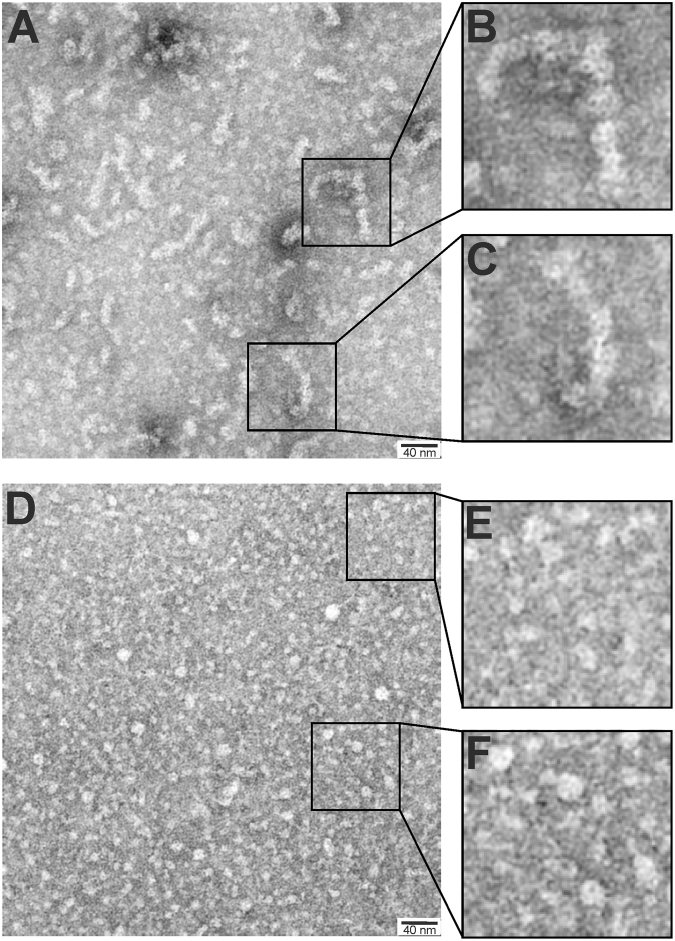
TEM images of Emp_FL_ (**A** to **C**) and Emp_2+3_ (**D** to **F**). The proteins are visible as light structures.

Thus, we analysed Emp_FL_ and Emp_2+3_ by TEM. The Emp_FL_ protein formed bead-like fibril structures of approximately 8 to 180 nm in thickness and 20 to 180 nm in length, which might be even longer due to coiling that was not clearly visible on the images (Fig. 6A). Zooming into such structures (Fig. 6B and C) revealed packages of smaller ‘donut’-like structures of approximately 8 nm in diameter (80 Å), which might represent the monomers. The N-truncated Emp_2+3_ protein did not form fibrils (Fig. 6D), but the ‘donut’-like structures could be observed in small packages of a few (2–4) units (Fig. 6E and F), indicating that the N-terminus is necessary for fibrillation.

The hydrodynamic diameter, as determined by the Nanosizer, was approximately 190 nm (±45 nm) for the main peak and 10.1 nm (±3.5 nm) for a low-molecular-mass fraction (Supplementary Figure S4). This finding supports the TEM results, but the polydispersity index of 0.62 ± 0.12 indicated a broad distribution of the Emp particles. Both parameters support the idea of a polymeric and fibrous tertiary structure of Emp.

### The protein model of Emp revealed structural similarities to leucine-rich repeat proteins

**Figure 7:**
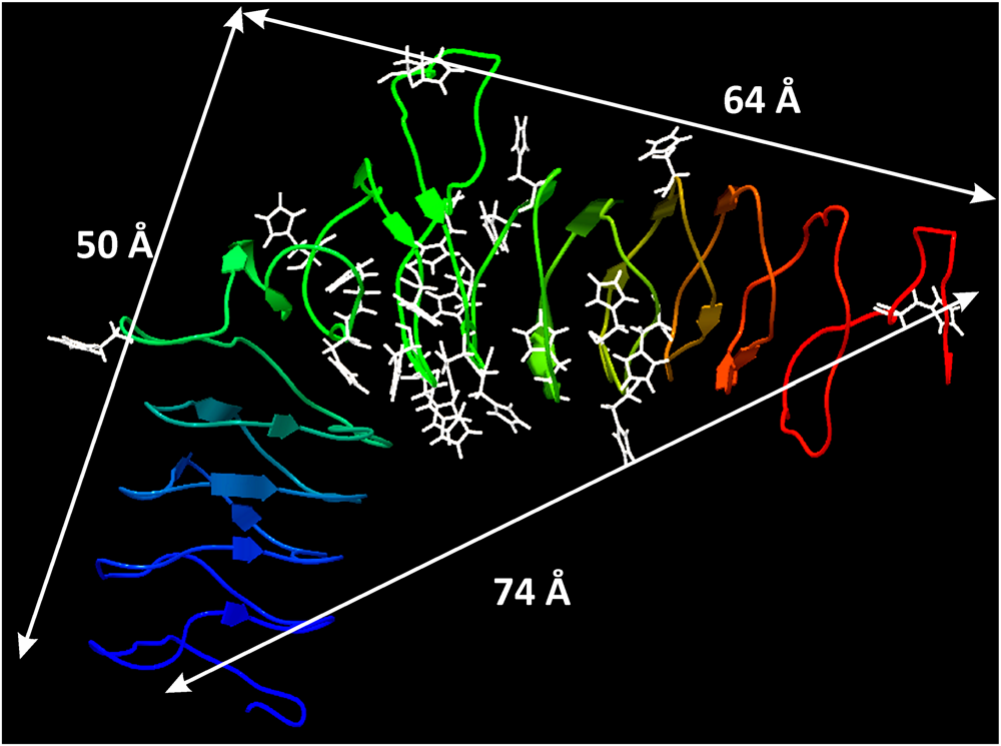
Predicted protein structure of Emp. The analysis was performed using the I-TASSER on-line server. The backbone is shown as a cartoon coloured from blue (N-terminus) to red (C-terminus); the histidine residues are represented as white sticks. The distances of the arms and the diagonal are indicated by arrows in Angstroms (Å).

Based on the Emp sequence, a possible protein structure of Emp was predicted using the I-TASSER on-line server. The analysis yielded four models, among which only one structure (Fig. 7) exhibited eligible scores: C-score = −2.95, TM-score = 0.38 ± 0.13 and root mean square deviation of atomic positions (RMSD) = 13.4 ± 4.1 Å. This predicted Emp structure (Fig. 7) showed a nearly 90° angled shape, with two arms of approximately 50 Å (5 nm, N-terminal) and 64 Å (6.4 nm, C-terminal) and a diagonal between both arms 73 Å (7.3 nm) in length. The primary structure contained short β-sheets and disordered structures, as predicted by CD spectroscopy. However due to the low C- and TM-scores, the overall folding of Emp remains unclear. The C-score is a confidence score for estimating the quality of predicted models by I-TASSER and typically ranges between −5 and 2; the higher the C-score, the higher is the confidence of the model. The template modelling (TM)-score and the RMSD are known standards for measuring structural similarity between two proteins with different tertiary structures. The TM-score ranges between 0 and 1, where 1 indicates a perfect match between two structures; the likelihood of similar folding increases when the TM-score exceeds 0.5^38^.

The top ten identified structural analogues, all exhibit an arc or horseshoe shape, were primarily different leucine-rich repeat (LRR) proteins and some eukaryotic receptors containing LRR motifs (for details, see Supplementary Table S1). The LRR domains are often associated with innate immunity in higher eukaryotes and can be found in mammalian Toll-like receptors (TLRs) and NOD-like receptors (NLRs), which sense pathogen-associated molecular patterns (PAMPs)^39^. Interestingly, the known Emp proteins do not contain noticeable amounts of leucine and no leucine repeats such as LRRs, but most of the histidines seem to be located in the concave face of the Emp-arc and might be co-responsible for the predicted shape. Histidines are known to be involved in interactions with different ligands, most often with cationic metals. However, due to very low C-scores, no eligible predictions can be made regarding the ligands based on the predicted structure.

## Conclusions

Our results suggested similar behaviours for Emp and Eap. *S*. *aureus* has a greater capacity to adhere to host tissue during inflammation. However, the roles of both proteins seem to be related to the late stage of infection. During the early stage of infection, many staphylococcal virulence factors, e.g. the staphylococcal protein A (SpA), interact with immunoglobulins inhibiting the adaptive host immune response. Staphylococcal virulence factors have also been shown to recruit neutrophils simultaneously inducing their apoptosis that triggers damage to host tissue due to enzymatic and acidic degradation of the ECMs^40,41^. This might enable interactions of Eap and Emp with matrix compounds that facilitate the access to deeper tissue for *S*. *aureus*. Furthermore, Eap contributes to the anti-inflammatory functions by blocking adhesion receptors of the leukocytes required for inflammatory infiltration of infected tissues protecting *S*. *aureus* from killing by leucocytes^42^. Thus, disintegrating suprastructures of the ECM are less suitable for infiltration by leukocytes, the directional migration of which depends on specific signaling molecules exposed by intact ECM.

**Figure 8:**
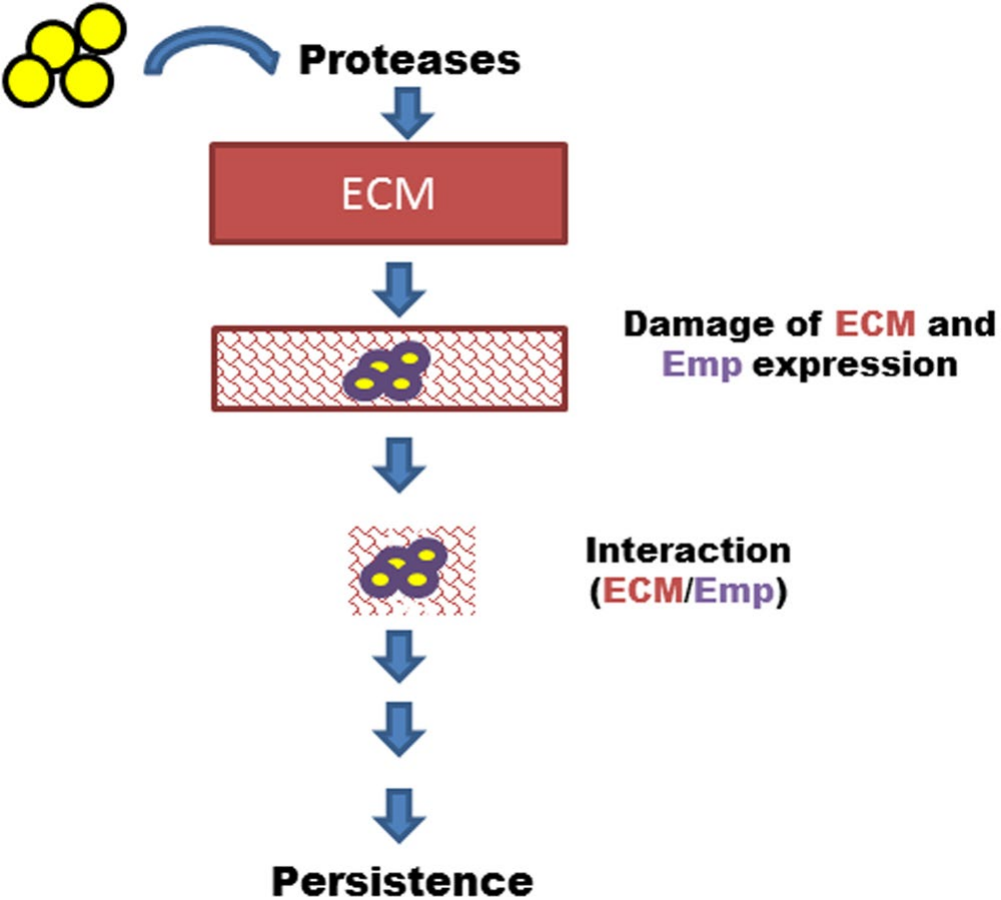
Hypothetical role of Emp during infection. *S*. *aureus* secretes proteases which disrupt the integrity of ECM. This process favours the exposition of epitopes and induces an interaction between Emp and ECM that contributes to staphylococcal persistence.

Emp exhibits higher expression in chronic osteomyelitis isolates^18^. Moreover, *S*. *aureus* requires Emp for staphylococcal accumulation which is the prerequisite for abscess formation and persistence in host tissues^19^. During infection, *S*. *aureus* induces the expression of proteolytic enzymes like V8, aureolysin to damage the ECM that favours the ability of *S*. *aureus* to develop deep tissue infections^43,44^. This mechanism facilitates the interaction between Emp and damaged ECM to promote *S*. *aureus* persistence (Fig. 8).

Emp seems to form fibrous structures at least 200 nm in length. The pili and fibers are typically found in bacterial adhesins^45^. Most fimbriae adhesins were found in Gram-negative bacteria, but many of them were also found in Gram-positive bacteria^46^. Pilin proteins serve a variety of functions, including adhesion to eukaryotic host cells, biofilm formation and horizontal gene transfer. Their polymerization permits extension and lengthening of the fiber, and their depolymerization results in pilus retraction^47^. However, the mechanisms known for fimbriae and pilins, which are multi-modular and involve several specific proteins in an energy-dependent manner, do not seem to apply to Emp. Emp correlates better with the protein ‘curli’ described for Gram-negative bacteria, which is also involved in adhesion to host components and invasion, cell aggregation and biofilm formation, and it may induce the inflammatory response^48^. In *E*. *coli*, the protein CsgA is also secreted into the environment and re-binds to its specific CsgB protein. Both proteins are encoded by the operon *csgAB*, which is divergently located in the *csgDEFG* operon; the second operon encodes additional proteins that are involved in curli assembling^48^. In contrast, Emp is encoded by a monocistronic gene and seems to directly re-bind to the *S*. *aureus* surface. A specific protein that is recognized by Emp has not been identified.

The adhesive properties of Emp are located in the C-terminus, which seems to be highly conserved within different staphylococcal species. The C-terminus also bears all of the conserved histidines that might be key residues for the adhesive characteristics of Emp. The N-terminus seems to be related to the polymerization of Emp to form a fibrillary structure. Emp showed higher adhesion to skin ECM than to cartilage or bone. These results suggest a role for Emp in wound infections, as previously described for Eap^49^. The fibre-like structure of Emp might also facilitate adherence of *S*. *aureus* to catheters and foreign material, resulting in biofilm and abscess formation^19,20^.The Emp monomer seems to exhibit a novel protein structure that is not related to any other known structure, but might be analogous to the LRR domains. We hypothesize that the high histidine content of the Emp-homologs could play a key role in their structure and function which is still elusive. It is also unclear how the fibre-like polymers are formed by these monomers and which lengths they may achieve *in vivo*. But this study provides the first insights into the structural properties of Emp, which might be a novel immune evasion mechanism of *S*. *aureus* and a potential target for the development of more efficient therapeutic alternatives to prevent or resolve staphylococcal infection.

## Materials and Methods

### Cloning of the Emp expression plasmids

The sequence encoding the full-length Emp protein (without the signal peptide) of *S*. *aureus* was amplified and cloned into the expression plasmid pQ30-UA (Qiagen, Hilden, Germany) to generate plasmid pQemp^10^. The truncated and recombined fragments of Emp (Emp_1_, Emp_2_, Emp_3_, Emp_1+2_, Emp_2+3_) were amplified by PCR using the sets of primers listed in Table 2. The PCR products were ligated into plasmid pQ30-UA (Qiagen) (Fig. 1) using T4 DNA Ligase (Thermo Fisher Scientific, Massachusetts, USA) according to the manufacturer’s protocol. The ligation mixture was transformed into freshly prepared competent *E*. *coli* TG1 cells, and transformants were selected on LB agar plates supplemented with 100 µg/ml ampicillin as described previously^10^. The plasmids were isolated using the QIAGEN Plasmid Mini Kit (Qiagen, Hilden, Germany) and were confirmed by sequencing at Eurofins Genomics (Ebersberg, Germany). Representative plasmids containing the different *emp* fragments were designated pQemp1, 2, 3, 1 + 2, and 2 + 3.

**Table 2:** Primers used in this study.

Name	Sequence 5′→3′	Position relative to ATG of Emp
EmpFor1UA	GCATCAGTGACAGAGAGTGTTGACAAA	+76
EmpRev1UA	ATTCACGCGATTATTGATTTCTTTGTA	+348
EmpFor2UA	TACAAAGAAATCAATAATCGCGTGAAT	+322
EmpRev2UA	ATGCGCGTGATATCCTGGAACTGCAAA	+648
EmpFor3UA	TTTGCAGTTCCTGGATATCACGCGCAT	+622
EmpRev3UA	TTATACTCGTGGTGCTGGTAAGCTACC	+1024

### Expression and purification of recombinant Emp protein forms

His-tagged Emp forms were expressed for 24 h in 2 L of LB broth supplemented with 1% lactose. The cells were harvested by centrifugation at 5,600 rpm and 4 °C for 10 min. The pellets were resuspended in 10 ml of lysis buffer (8 M urea, 0.5 M NaCl, 10 mM β-mercaptoethanol, 5% glycerol, and 1% Triton X-100 in 50 mM sodium phosphate buffer, pH8.0) and were sonicated using a Bandelin Sonopuls GM 2200 or a Bandelin Electronic UW 2200 (Bandelin Electronic GmbH & Co. KG, Berlin, Germany) for 6 min on ice. After centrifugation at 13,000 rpm at 4 °C for 30 min, the supernatants were mixed with 3 ml of Ni-nitrilotriacetic acid (NTA) affinity resin (Qiagen, Hilden, Germany) and shaken at 33 rpm for 2 h at 4 °C on a Stuart^™^ SRT6 roller mixer (Sigma, Missouri, USA). Subsequently, the mixtures were applied to columns, and the flowthroughs were discarded. Each column was washed three times with 10 ml of lysis buffer containing 30 mM imidazole and eluted with 3 ml of lysis buffer containing 250 mM imidazole. The purity of the eluted proteins was analysed by SDS-electrophoresis on 12% acryl amid gels and western blot analysis as described previously^10^. The proteins were solubilized sequentially by dialysis against buffer A1 (4 M urea, 0.5 M NaCl, and 10 mM β-mercaptoethanol in 50 mM phosphate buffer, pH8.0), buffer B1 (1 M urea, 0.5 M NaCl, and 10 mM β-mercaptoethanol in 50 mM sodium phosphate buffer, pH8.0) and finally buffer C1 (0.25 M NaCl in 50 mM sodium phosphate buffer, pH8.0). The protein was quantified using a NanoDrop (PeqLab, Erlangen, Germany) at 280 nm by applying the specific extinction coefficient (Supplementary Table S2).

### Sequence analysis

All sequence analyses were performed using CLC Main Workbench 7.0.2 (Qiagen). The secondary structure of the recombinant (His-tagged) Emp protein sequence was predicted using the Hidden Markov Model (HMM) provided by the CLC Main Workbench. Multiple alignments were performed by applying the Clustal Omega algorithm^50^ for multiple sequence alignments. Phylogenetic analysis was performed based on maximum likelihood phylogeny tree construction with 100 bootstrap replicates.

The Emp sequence was compared against the NCBI database using the BLAST platform and the blastp tool to identify related proteins.

### Extraction of matrix suprastructures

Fragments of suprastructural aggregates from human skin, bone and articular cartilage were obtained as described previously^16,18^. Briefly, slices of cartilage were homogenized twice using a Polytron in 15 volumes of PBS (150 mM NaCl in 2 mM sodium phosphate buffer, pH7.4) containing a mixture of protease inhibitors (0.1 mM phenylmethylsulforyl fluoride (PMSF), 0.1 M 6-aminocaproic acid, 5 mM benzamidine and 5mM N-ethylmaleimide (NEM)), and the homogenates were cleared by centrifugation at 4800 rpm for 6 min. This procedure was repeated twice. The supernatants containing suprastructural cartilage aggregates were used for adherence analysis. The epidermal layer of the skin samples was removed after treatment with 0.05 M Tris-HCl, pH7.4, containing 1 M NaCl for 4 h, and the dermal layer was frozen in liquid nitrogen. A 200-µm-thick layer was removed from the dermal surface by a dermatome and was repeatedly homogenized in PBS containing protease inhibitors (0.1 mM PMSF, 0.1 M 6-aminocapron acid, 5 mM benzamidine and 5 mM NEM). Between each homogenization step, tissue debris was removed by centrifugation at 4,800 rpm for 6 min. The supernatants containing supramolecular dermal aggregates were used for adherence analysis.

Fragments of suprastructural aggregates from bone were extracted from human hips recovered from joint replacements. The hips were carefully cleaned of adhering connective tissue and bone marrow followed by demineralization using sodium-EDTA (20 g/l, pH7.4) for four weeks, changing the demineralizing buffer every 3 days. After demineralization, the bone was mechanically disrupted into small pieces and homogenized in PBS (150 mM NaCl in 2 mM sodium phosphate buffer, pH7.4) containing a mixture of protease inhibitors (0.1 mM PMSF, 0.1 M 6-aminocaproic acid, 5 mM benzamidine and 5 mM NEM) using a Polytron homogenizer (Kinematica, Luzern, Switzerland). The obtained homogenates were clarified by centrifugation at 4,800 rpm for 6 min and were used for the experiments.

### Adherence assays with the Emp forms

Polystyrene microtiter plates were coated with 5 μg/ml of human skin, cartilage or bone fragment or with 0.25 μg/well of ECM components collagen I (Cn I), fibronectin (Fn), fibrinogen (Fg) or vitronectin (Vn) in 0.1 M Na_2_CO_3_ overnight at 4 °C. After washing with PBST (137.93 mM NaCl, 2.67 mM KCl, 1.47 mM KH_2_PO_4_, 8.06 mM Na_2_HPO_4_ × 7 H_2_O and 0.2% Tween 20, pH7), the wells were blocked by incubation with protein-free blocking buffer (Pierce, Rockford, IL USA) for 1 h at room temperature (RT). The wells were washed with PBST, and 50 µl of different concentrations of Emp_FL_ and Emp fragments (0, 0.01, 0.02, 0.03, 0.04, 0.05, 0.075, 0.1, 0.25, 0.5, 0.75, 1, and 1.5 µM) were added to each well for the concentration-dependent assays. Other binding assays were performed with 1 µM of the Emp_FL_ and Emp forms. The plate was incubated at RT for 1 h with shaking at 70 rpm. The wells were washed with PBST, and 50 µl of a 1:1000 dilution of polyclonal anti-Emp IgG (Squarix Biotechnology, Germany) was added to each well followed by re-incubation of the plate at RT for 1 h with shaking at 70 rpm. The wells were washed with PBST, and 50 µl of a 1:2000 dilution of alkaline phosphatase (AP)-conjugated anti-rabbit goat IgG (Dako, Eching, Germany) was added to each well followed by shaking at 70 rpm for 1 h at RT. The plates were washed three times with PBST, and 50 µl of AP-buffer (1 mM MgCl_2_, in 0.1 M Tris/HCl pH9.5) supplemented with ready-to-use Alkaline Phosphatase Yellow (pNPP) substrate (Sigma-Aldrich, Missouri, USA) was added to each well. The colour changes were measured at 405 nm after a 30 min incubation at RT using an iMark microplate reader (Bio-Rad, Hercules, California, USA).

### Immunogold transmission electron microscopy

Aliquots of suprastructural fragments were absorbed to formvar/carbon nickel grids, washed with PBS and treated for 30 min with 2% (w/v) dried skim milk in PBS for cartilage and bone or 2% (w/v) bovine serum albumin (BSA) in PBS for skin. This procedure was followed by incubation with recombinant Emp (full-length or fragments) in PBS at different concentrations for 1 h at RT. After several steps of washing with PBS, the grids were allowed to react with a rabbit polyclonal antibody against Emp diluted 1:100 in 0.2% (w/v) dried skim milk or PBS containing 2% (v/v) BSA-c (Aurion, Wageningen, Netherland) and 0.025% (v/v) Tween 20 (blocking solution). After washing with PBS, the grids were placed on drops of PBS containing goat antibodies against rabbit immunoglobulins conjugated to colloidal gold particles (Jackson Immuno Research Laboratories) diluted 1:30 in 0.2% dried skim milk or blocking solution. In some experiments, the suprastructural fragments were treated with purified collagenase (CLSPA; Worthington, New Jersey, USA) for 2 h at 37 °C. Finally, the grids were washed five times with distilled water and negatively stained with 2% (w/v) uranyl acetate for 10 min. Electron micrographs were obtained with a Philips EM-410 electron microscope at 60 kV.

### Transmission electron microscopy (TEM)

Carbon-coated grids (400 meshes) (Quantifoil, Großlöbichau, Germany) were hydrophilized by glow discharge at low pressure in air. Subsequently, 20 μl a solution of purified Emp_FL_ (3.1nmol) or Emp_2+3_ (2.4nmol), was adsorbed onto the hydrophilic grids for 1 min. The grids were washed twice with drops of distilled water and were stained with a drop of 2% uranyl acetate in distilled water. The samples were analysed using a Zeiss EM902A electron microscope (Carl Zeiss AG, Oberkochen, Germany) operated at an acceleration voltage of 80 kV. Images were recorded with a FastScan-CCD camera at 1024 × 1024 pixels (TVIPS, Munich, Germany).

### Circular dichroism spectroscopy (CD)

Chiral macromolecules, such as proteins differentially absorb left- and right-handed circularly polarized light (L-CPL or R-CPL). These differences can be visualized by circular dichroism (CD) spectroscopy. Depending on whether L-CPL or R-CPL is absorbed to a greater or to a lesser extent, the CD signal can be positive or negative. The electron transitions events of the chiral amide groups, which can be observed at the far ultraviolet (UV) region (below 260 nm), are highly influenced by their surrounding geometries (ϕ and ψ torsion angles) of the polypeptide backbone. Thus, the resulting CD spectra are specific for the secondary structures of the protein (α-helices, β-turns, and natively disordered)^51^.

For CD spectroscopy, the proteins were finally dialysed against 250 mM NaF in 50 mM sodium phosphate buffer, pH7. CD measurements were performed on a Jasco J-815 spectropolarimeter in 1mm quartz cuvettes (Hellma, Müllheim, Germany). CD spectra were recorded between 185 and 320 nm. Twenty scans for the analysis of protein secondary structure were accumulated and averaged. The buffer reference spectra (250 mM NaF in 50 mM sodium phosphate buffer, pH7) were used as baselines and were subtracted from the CD spectra of the samples. Signal intensities were expressed in millidegrees [θ] and converted to the mean molar ellipticity per residue [θ]_MRE_ in (deg cm^2^ dmol^−1^ residue^−1^) according to equation (1), where ‘N’ represents the number of amino acid residues, ‘d’ is the path length of the cell in cm, ‘c’ is the concentration in g/L of protein, and M is the molecular mass in Da.

1 $${[\theta ]}_{{\rm{MRE}}}=\frac{\theta \ast M}{10\ast {\rm{c}}\ast {\rm{d}}\ast {\rm{N}}}$$

The analysis of protein secondary structures was performed on the DichroWeb server^52^ using SELCON3^53–55^, CONTIN^56,57^, CDSSTR^55,58^ algorithms and different reference datasets. As CD data were reliable only above 190 nm, datasets 3, 4, 6, 7, SP175 and SMP180 could be used. The normalized root mean square deviation (NRMSD) was used as a goodness-of-fit parameter for each deconvolution output^52^.

### Hydrodynamic protein size measurements

The photon correlation spectroscopy (PCS) uses the quasi-elastic/dynamic light scattering of small particles in suspension or polymers in solution based on the principle that smaller particles move with higher velocity than larger particles. Thereby, the time-dependent Brownian motion of the particles/polymers is detected as speckle patterns and the hydrodynamic diameter information of the particles is derived from the autocorrelation $\wp ={q}^{2}{D}_{t}$ of the intensity traces of each spot, where $\wp$ is the decay rate, q is the wave vector and D_t_ is the diffusion coefficient that can be used to calculate the hydrodynamic diameter by Stokes-Einstein equation.

Applying the PCS the hydrodynamic diameter distribution and the polydispersity index of 12.94 µM Emp_FL_ in dialysis buffer (50 mM sodium phosphate, pH7, 250 mM NaF) were measured. The experiments were performed in a low-volume cuvette (ZEN 0112, Brand, Wertheim, Germany) using a Zetasizer Nano ZS (Malvern Instruments, Herrenberg, Germany) at 25 °C and at a scattering angle of 173°. It total, six measurements were collected and evaluated using Zetasizer software (version 5.03, Malvern Instruments) and applying the refractive index (1.33) and viscosity (0.9872mPa*s) of the dialysis buffer. Data are expressed as the means with standard deviations.

### Fourier transform infrared spectroscopy (FTIR)

In the Fourier transform infrared spectroscopy (FTIR), all of the infrared (IR) frequencies are assessed simultaneously by splitting the IR light into two beams; one with a fixed wave length, the other with is constantly length changing wave length. Both beams are merged to an interfering beam to with the sample is exposed resulting in an interferogram. The infrared frequencies encoded in an interferogram must be deconvoluted by the Fourier transformation yielding a frequency spectrum. The IR spectrum is a kind of molecular finger print of biomacromolecules. In contrast to other techniques, FTIR requires only small amounts of proteins (1 mM) in variable deuterium water allowing high quality spectra independent of the background fluorescence or light scattering. Additionally, FTIR is not limited to specific protein sizes. Because of the repeating units in proteins, nine characteristic IR absorption bands (amides A, B and I–VII) can be measured. Thereby, the amide I bands (1,700–1,600 cm^−1^) are the most sensitive vibrational bands of the polypeptide backbone, which gives specific peaks depending of the secondary structural components^59^.

For FTIR spectroscopy, the proteins were dialyzed stepwise against buffer A2 (50% D_2_O and 0.25 M NaCl in 50 mM sodium phosphate buffer, pH8.0), buffer B2 (75% D_2_O and 0.25 M NaCl in 50 mM sodium phosphate buffer, pH8.0) and finally buffer C2 (100% D_2_O and 0.25 M NaCl in 50 mM sodium phosphate buffer, pH8.0). Infrared (IR) spectra were recorded with a Nicolet Magna 750FTIR spectrometer (Thermo Fisher Scientific, Waltham, Massachusetts, USA) equipped with a DTGS detector. The standard proteins myoglobin, concanavalin A and lysozyme were prepared in buffer C2 at concentrations of 3 µg/µl, which corresponded to the estimated concentration of Emp. Forty-five microliters of the sample solution was placed in a demountable press-on cell for analysis of the liquids with calcium fluoride windows and a 100 µm Teflon spacer (Pike Technologies, Wisconsin, USA). A total of 256 scans were averaged per interferogram at a spectral resolution of 4 cm^−1^. The total spectral range was 950–4000 cm^−1^. The IR spectra were corrected for water vapour with OMNIC software version 9 (Thermo Fisher Scientific). The IR spectra were baseline-corrected and normalized in the spectral range from 1780–1540 cm^−1^ for comparison of amide I bands.

### Prediction of the Emp structure

The I-TASSER (Iterative Threading ASSEmbly Refinement) on-line server (http://zhanglab.ccmb.med.umich.edu/I-TASSER/) was used to predict the Emp structure without any specific assumptions. In I-TASSER, full-length structural models are constructed by iterative fragment assembly simulations utilizing structural templates identified in the protein data bank using multiple threading alignments^60,61^. Furthermore, the constructed model is compared to structures of known proteins (top ten structural analogues), offering insights into funicular properties^62^. The most fitting model was visualized and further analysed using CLC Main Workbench.

### Curve fittings and statistics

Statistical analyses and curve fittings were performed using GraphPad Prism version 4.00 (GraphPad Software, San Diego California USA, www.graphpad.com). The binding constants of the Emp forms were calculated by applying the one-site binding model according to equation (2); where X represents the concentration of the ligand and Y is the specific binding signal that increases to a maximum plateau value Y_max_. The K_D_ is the equilibrium dissociation constant, expressed in the same units as the X-axis (concentration in µM). When the drug concentration equals K_D_, half the binding sites are occupied at equilibrium.

2 $${\rm{Y}}=({{\rm{Y}}}_{{\rm{\max }}}\ast {\rm{X}})/({{\rm{K}}}_{{\rm{D}}}+{\rm{X}})$$

Differences in the binding of the Emp_FL_ and Emp fragments to the suprastructures were assessed by one-way ANOVA and Dunnett’s multiple comparison test or by two-way ANOVA and the Bonferroni post-test (two-tailed, 95% confidence intervals). Significance was assumed with p-values ≤ 0.05.

### Ethical statements

ECM preparations and experimental protocols from human skin and cartilage and human bone tissue were approved by the ethics committee of the University of Muenster (Ethikkomission der Ärztekammer Westfalen-Lippe und der Medizinischen Fakultät der Wesfälischen Wilhelms-Universität, Münster), Az. 2009-442-f-s and Az. 2010-155-f-s, respectively. All experiments were performed in accordance with relevant guidelines and regulations and approved by the universities of Muenster and Jena, Germany. The informed consent was obtained from all participants.

## Electronic supplementary material

Supplementary material
