# Author Correction: The *Staphylococcus aureus* extracellular matrix protein (Emp) has a fibrous structure and binds to different extracellular matrices

**DOI:** 10.1038/s41598-018-26315-6

**Published:** 2018-05-25

**Authors:** Jennifer Geraci, Svetlana Neubauer, Christine Pöllath, Uwe Hansen, Fabio Rizzo, Christoph Krafft, Martin Westermann, Muzaffar Hussain, Georg Peters, Mathias W. Pletz, Bettina Löffler, Oliwia Makarewicz, Lorena Tuchscherr

**Affiliations:** 1 Institute of Medical Microbiology, Jena University Hospital, Jena, Germany; 2 Center for Infectious Diseases and Infection Control, Jena University Hospital, Jena, Germany; 3 Center for Sepsis Control and Care (CSCC), Jena University Hospital, Jena, Germany; 4 InfectoGnostics Research Campus, Jena, Germany; 5 Institute of Medical Microbiology, Münster University Hospital, Münster, Germany; 6 Institute of Experimental Musculoskeletal Medicine, Münster University Hospital, Münster, Germany; 7 Institute of Molecular Science and Technologies (ISTM-CNR), Milano, Italy; 8 Organic Chemistry Institute and CeNTech, Westfälische Wilhelms-Universität Münster, Münster, Germany; 9 Leibnitz Institute for photon technologies, Jena, Germany; 10 Center for electron microscopy Jena University Hospital, Jena, Germany

Correction to: *Scientific Reports* 10.1038/s41598-017-14168-4, published online 20 October 2017

In the original version of this Article, Bettina Löffler and Lorena Tuchscherr were incorrectly affiliated with ‘Center for Infectious Diseases and Infection Control, Jena University Hospital, Jena, Germany’. The correct affiliation is listed below:

Center for Sepsis Control and Care (CSCC), Jena University Hospital, Jena, Germany

In addition, this affiliation was omitted for Christine Pöllath.

This new affiliation is listed as Affiliation 3 and, as a result, Affiliations 3 – 9 in the original version of this Article are now Affiliations 4 – 10.

These errors have now been corrected in the PDF and HTML versions of the Article and in the accompanying Supplementary Information.

