# Supplementary material for: The Staphylococcus aureus extracellular matrix protein (Emp) has a fibrous structure and binds to different extracellular matrices
Source: Sci Rep. 2017 Oct 20;7:13665. doi: 10.1038/s41598-017-14168-4 (PMC5651841; doi:10.1038/s41598-017-14168-4)
Supplement: Supplementary file 1 — Supplementary material [file 41598_2017_14168_MOESM1_ESM.docx]

**Supplementary Material**

**“The *Staphylococcus aureus* extracellular matrix protein (Emp) has a fibrous structure and binds to different extracellular matrices”**

**Authors**

Jennifer Geraci^1^, Svetlana Neubauer^2,4^, Christine Pöllath^1,3^, Uwe Hansen^6^, Fabio Rizzo^7, 8^, Christoph Krafft^9^, Martin Westermann^10^, Muzaffar Hussain^5^, Georg Peters^5^, Mathias W. Pletz^2,4^, Bettina Löffler^1,3^, Oliwia Makarewicz^2,4§^, and Lorena Tuchscherr^1,3§^

1 Institute of Medical Microbiology, Jena University Hospital, Jena, Germany

2 Center for Infectious Diseases and Infection Control, Jena University Hospital, Jena, Germany

3 Center for Sepsis Control and Care (CSCC), Jena University Hospital, Jena, Germany

4 InfectoGnostics Research Campus, Jena, Germany

5 Institute of Medical Microbiology, Münster University Hospital, Münster, Germany

6 Institute of Experimental Musculoskeletal Medicine, Münster University Hospital, Münster, Germany

7 Institute of Molecular Science and Technologies (ISTM-CNR), Milano, Italy

8 Organic Chemistry Institute and CeNTech, Westfälische Wilhelms-Universität Münster, Münster, Germany

9 Leibnitz Institute for photon technologies, Jena, Germany

10 Center for electron microscopy Jena University Hospital, Jena, Germany

**Corresponding Authors:** Lorena Tuchscherr and Oliwia Makarewicz

§ These authors contributed equally to this work.

**Supplementary Table S1:** The top ten identified structural analogs of the predicted Emp-structure model by I-TASSER [[1-3](#_ENREF_1)].

| Ranking^1^ | PDB Hit | TM-score | RMSD^a^ | IDEN^a^ | Cov | Protein |
| --- | --- | --- | --- | --- | --- | --- |
| 1 | 4h09A | 0.894 | 1.66 | 0.118 | 0.943 | leucine-rich repeat protein (EUBVEN_01088) from *Eubacterium ventriosum* ATCC 27560 |
| 2 | 4cp6A | 0.806 | 2.81 | 0.094 | 0.908 | pneumococcal vaccine antigen PcpA |
| 3 | 4gt6A | 0.731 | 2.40 | 0.070 | 0.812 | leucine rich cell surface protein (FAEPRAA2165_01021) from *Faecalibacterium prausnitzii* A2-165 |
| 4 | 4bv4R | 0.678 | 4.21 | 0.056 | 0.904 | cytokine Spatzle from *Drosophila melanogaster* that binds to a Toll-receptor |
| 5 | 4ecnA | 0.675 | 4.38 | 0.070 | 0.908 | leucine-rich repeat protein (BT_0210) from *Bacteroides thetaiotaomicron* VPI-5482 |
| 6 | 2id5D | 0.670 | 4.31 | 0.053 | 0.901 | leucine-rich repeat ectodomain of transmembrane protein Lingo-1 in *Homo sapiens*, |
| 7 | 4z5wA | 0.668 | 4.38 | 0.060 | 0.908 | plant peptide hormone receptor of *Daucus carota* |
| 8 | 4z0cA | 0.668 | 4.65 | 0.072 | 0.930 | Toll-like receptor 13 of *Mus musculus* |
| 9 | 3rizA | 0.666 | 4.33 | 0.053 | 0.901 | plant steroid receptor BRI1 ectodomain *Arabidopsis thaliana* |
| 10 | 4kt1A | 0.666 | 4.35 | 0.067 | 0.895 | R-spondin 1 containing leucine-rich repeats |

^1^The ranking of proteins is based on TM-score of the structural alignment between the query structure and known structures in the protein data base (PDB) library. RMSD^a^ is the RMSD between residues that are structurally aligned by TM-align. TM-score is the template modeling score and ranges between 0 and 1, where 1 indicates a perfect match between two structures; and the likelihood of similar folding increases when TM-score is higher than 0.5 [[4](#_ENREF_4)]. IDEN^a^ is the percentage sequence identity in the structurally aligned region. Cov represents the coverage of the alignment by TM-align and is equal to the number of structurally aligned residues divided by length of the query protein.

**Supplementary Table S2.** Emp fragments analyzed in this study, their specific molecular masses (MM), as well as predicted specific extinction coefficients (ɛ, for reduced cysteine) and isoelectric points (IEP).

| **Protein** | **MM in kDa** | **ɛ in L/(mol*cm)^-1^** | **IEP** |
| --- | --- | --- | --- |
| Emp_1_ | 12.306 | 2,560 | 8.89 |
| Emp_2_ | 14.999 | 6,400 | 10.04 |
| Emp_3_ | 17.522 | 16,700 | 10.04 |
| Emp_1+2_ | 23.789 | 7,680 | 9.82 |
| Emp_2+3_ | 29.157 | 21,820 | 10.16 |
| Emp_FL_ | 37.947 | 23,040 | 9.99 |


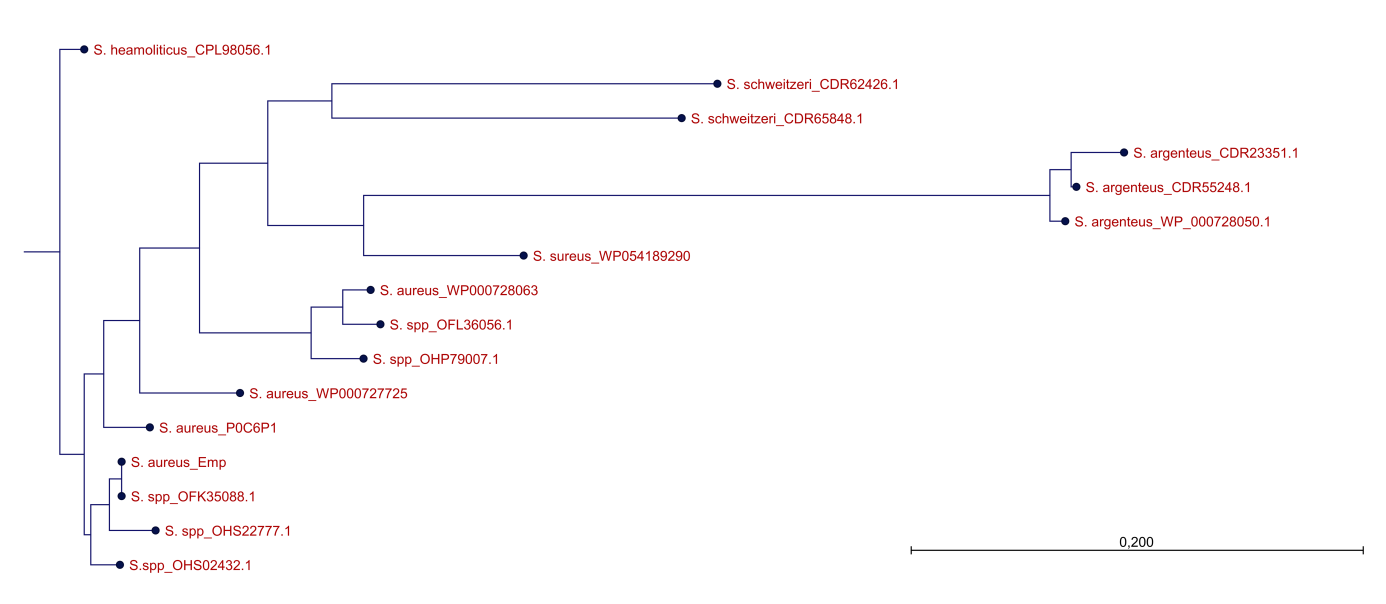


**Supplementary Figure S1.** Phylogenetic relationship of Emp proteins from *S. aureus*, *S*. spp, *S. heamoliticus*, *S. schweitzeri*, and *S. argenteus*. The analysis was performed based on the maximum likelihood phylogeny tree construction with 100 bootstrap replicates.


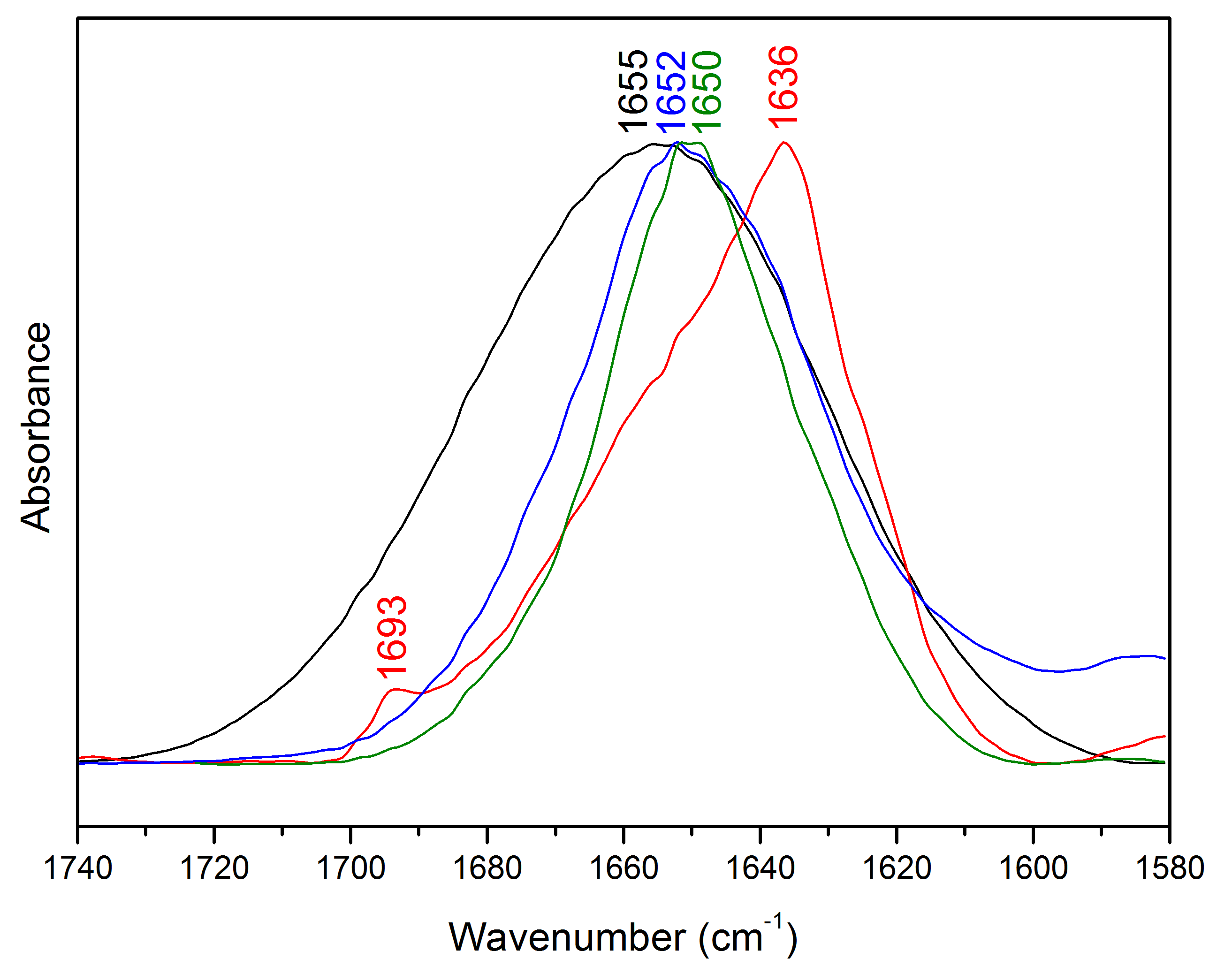


**Supplementary Figure S2** IR spectra of the amide I bands of concanavalin A (red trace), myoglobin (green), lysozyme (blue) and Emp (black) from 1740 to 1580 cm^-1^. Spectra have been corrected for baseline and water vapor, and normalized for display.


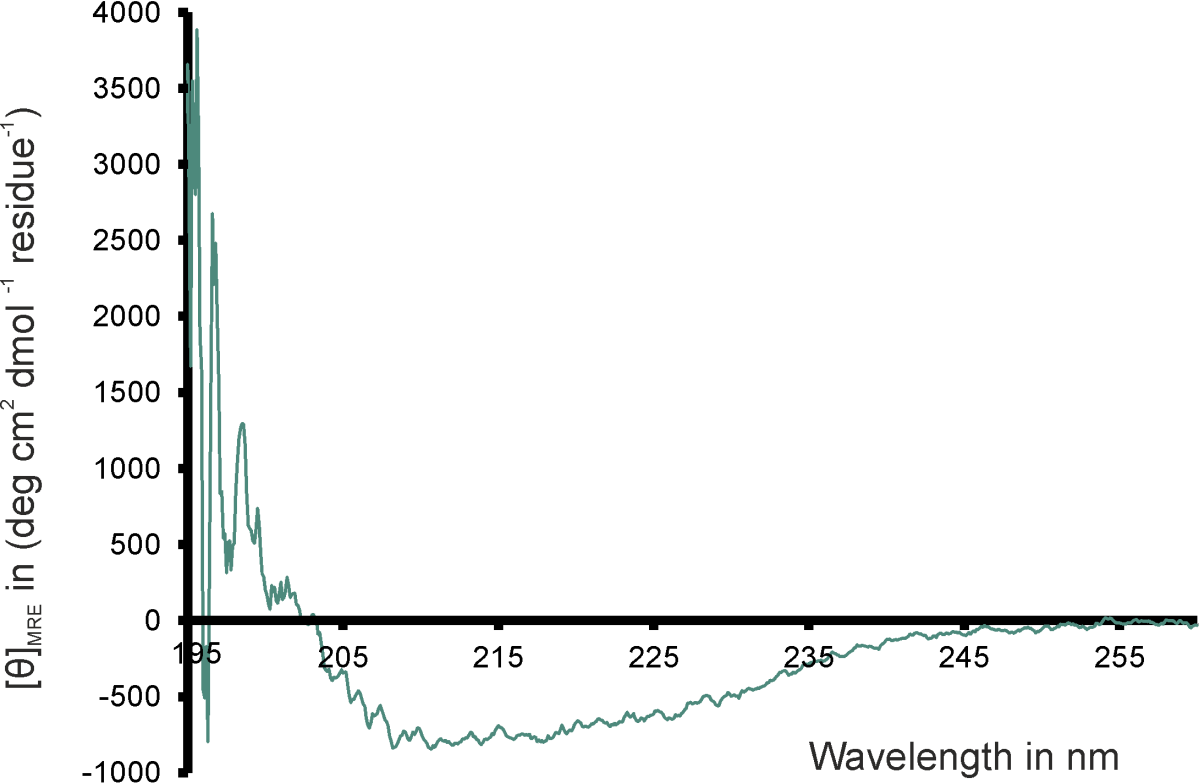


**Supplementary Figure S3.** Far-UV CD-spectrum of Emp_FL_. 20 measurements were accumulated. The signal quality below 195 nm was too low for deconvolution. Signal intensities were expressed as the mean molar ellipticity per residue [θ]_MRE_ in millidegrees [θ] . The concentration of Emp_FL_ was 107.5 µg/ml and 336 amino acid residues were considered.


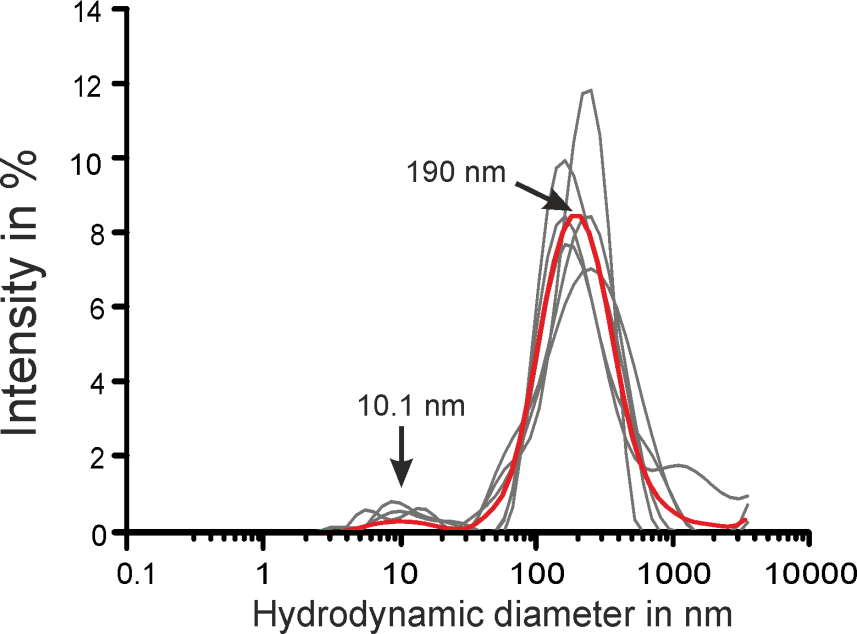


**Supplementary Figure S4.** Distribution of the hydrodynamic diameters (nm) of Emp_FL_. The averaged hydrodynamic diameter (red line) was calculated from 6 measurements (grey lines).

**Supplementary References**

1. Yang, J. and Y. Zhang, *I-TASSER server: new development for protein structure and function predictions.* Nucleic Acids Res, 2015. **43**(W1): p. W174-81.

2. Roy, A., A. Kucukural, and Y. Zhang, *I-TASSER: a unified platform for automated protein structure and function prediction.* Nat Protoc, 2010. **5**(4): p. 725-38.

3. Yang, J., A. Roy, and Y. Zhang, *Protein-ligand binding site recognition using complementary binding-specific substructure comparison and sequence profile alignment.* Bioinformatics, 2013. **29**(20): p. 2588-95.

4. Zhang, Y. and J. Skolnick, *TM-align: a protein structure alignment algorithm based on the TM-score.* Nucleic Acids Res, 2005. **33**(7): p. 2302-9.
